# Effects of personalized invitation letters on research participation among general practitioners: a randomized trial

**DOI:** 10.1186/s12874-021-01447-y

**Published:** 2021-11-13

**Authors:** Patrick Hennrich, Christine Arnold, Michel Wensing

**Affiliations:** Department of General Practice and Health Services Research, Heidelberg University Hospital, Im Neuenheimer Feld 130.3, 69120 Heidelberg, Germany

**Keywords:** Personalization, Response rate, General practitioners, Ambulatory health care, Invitation letter, Recruitment

## Abstract

**Background:**

Participation of general practitioners is crucial for health care studies. However, recruiting them is an ongoing challenge and participation rates of general practitioners around the globe are often low. One feasible and cost-efficient approach to potentially enhance participation rates among general practitioners are personalized invitation letters, since they may increase one’s attention to and appreciation of a study. Still, evidence whether this method actually affects participation is scarce and ambiguous in relation to physicians.

**Methods:**

We undertook a randomized trial in a sample of general practitioners from three German states in the context of a large, observational study on physicians’ coordination and uptake of recommended cardiovascular ambulatory care. An intervention group (*n* = 757 general practitioners) received a personalized invitation to participate in the observational study, the control group (*n* = 754 general practitioners) received a generic invitation. Both groups were blinded to group assignment. Eventual participation rates as well as the number and types of responses overall were compared between arms. Besides the main intervention, sociodemographic and geographical context factors were considered as well.

**Results:**

The overall participation rate among physicians was 2.6% (2.8% in the intervention group and 2.4% in the control group). No statistically significant effect of personalization on participation of physicians was found (relative risk to participate when receiving a personalized invitation of 1.17 [95%-CI: 0.62, 2.21]). However, the number of responses to the invitation varied significantly between the geographical regions.

**Conclusions:**

Personalization of first written contact alone did not improve research participation among general practitioners, which was overall very low.

**Trial registration:**

The study in which the trial was embedded has been registered prospectively at the German Clinical Trials Register (DRKS) under registration number DRKS00019219.

**Supplementary Information:**

The online version contains supplementary material available at 10.1186/s12874-021-01447-y.

## Introduction

The recruitment of physicians for scientific studies is crucial for health research. However, recruiting physicians for studies is a major challenge [1] as physician participation rate tends to be low. For instance, only around 3–4% of general practitioners (GPs) responded to study invitations in Germany, when lists of the ‘Kassenärztliche Vereinigung’ (association of statutory health insurance physicians) were used [2]. In April 2020, a large German funder of health services research had 379 studies listed [3] of which several reported problems achieving their calculated numbers of participants not only among patients, but among physicians as well [4]. The low participation of physicians in research has an impact on the validity and generalizability of the study findings [5].

For GPs, crucial barriers to participate in studies include high workload, practice staff that makes the physician inaccessible [6] and a perceived incompatibility between the physician role and participation in scientific research [7]. Research further showed that there is little interest in research among many GPs, because they perceive little relevance and were not involved in the study design [6, 7]. In some countries, practice networks for research in primary care have been established to facilitate scientific research in ambulatory care settings [8]. In Germany, some research networks for primary care research have been initiated as well, but most are at an early stage [2]. Other strategies have targeted the recruitment procedure itself to tackle the problem of low recruitment rates. In the social sciences, extensive methodological analyses and instructions on how to perform (mail) surveys are available, such as the prominent 1978 work by Don Dillman on the “Total Design Method” which has been reissued multiple times since then [9]. Potential approaches to increase response, respectively participation rates include, to name just a few, various kinds of incentives (monetary or not), personalized correspondence and reminders for non-responders. However, this research is mainly focused on surveys among the general public. It cannot be assumed that these methods can be directly generalized and applied to studies with physicians, especially since these presumably receive notably more invitations to participate in studies than the general public and hence might have a different attitude towards research per se.

The recruitment of individuals in health research has been extensively studied. A Cochrane review from 2009 of 513 randomized trials on paper-based as well as electronic surveys showed positive effects for, among others, (monetary) incentives, personalized questionnaires, first class mailing, short questionnaires, relevant topics and hand-written addresses [10]. The majority of studies focused on patients and various groups of health service providers. In 2014, a review on recruitment strategies purely for GPs by Pit et al. [11] showed that (especially monetary) incentives, postal surveys, mixed survey modes, pre-contacts as well as time and type of letter were positively associated with participation rate. Overall, these studies showed mixed effects on participation rates, which implies high uncertainty regarding the effectiveness of any particular strategy.

A relatively simple, low-cost approach to enhance participation is the use of a personalized letter in the first mailout to potential study participants. This may increase attention and appreciation of the invitation to participate in the study among the individuals who are addressed. Additionally, possible effects of personalization on response rates are not only relevant from a methodological point of view, but also regarding economic aspects. Manufacturing generic documents for a study is less time-consuming and eventually cheaper than a personalized letter for each potential participant. If positive personalization effects do in fact exist, their advantages regarding response rates may outweigh the additional effort. However, if no effects exist, personalization of documents might be a waste of time and money.

Studies in healthcare showed that personalized mailout may increase the participation rate, but the impact ranged from no effect to a modest effect (odds ratio (OR) 1.73 [95%-confidence interval (CI): 1.04, 2.87]) [11]. Additionally, different personalization effects were found depending on the number of mailouts that had been conducted – the authors of the review speculated that this might have been connected to the fact that various personalization methods were used [11]. Pit et al.’s results on personalization are similar to the ones found in the Cochrane review mentioned above where, including all stages of mailing out questionnaires, the total odds ratio of responding to personalized vs. less personalized documents were 1.14 [95%-CI: 1.07, 1.22] [[10], p. 323-24].

Outside the health domain, the impact of personalization has been, e.g., supported by a web-based survey study among students [12]. However, a more recent study on online-surveys from 2016 was not able to find a positive effect of personalized invitation letters on response rate [13]. Similar results were found in Italy for study invitations via text message – the authors observed a higher response rate in the intervention group receiving a personalized message, but the difference was not significant compared to the control group [14]. In 2018, a study conducted in Wisconsin showed no significant effects of personalization on response rates either – here, on the contrary, slightly higher participation occurred in the control group who received a generic letter. Furthermore, the authors showed a significant effect of personalization on item nonresponse, with participants who received a personalized letter having a higher rate of item nonresponse than those who received a generic letter [15]. Regarding the latter, it is already known from cross-sectional surveys that a personal cover letter may not only influence participation rate, but that it may also raise doubts about anonymity and reduce trust among potential study participants [16].

The studies presented show that the evidence on the effects of personalization strategies to increase recruitment rates in- and outside the medical domain is mixed. Not all studies on personalized documents were conducted among physicians or within the field of medicine – as we pointed out, one can assume differences between studies including physicians and studies including the general public. Furthermore, the definition of personalization may vary between studies: A personalized letter, e.g., might involve a personal address, individual tailoring of the content to the recipient, hand-written content, hand-signed content, referrals to previous studies the recipient participated in, a hand-stamped envelope instead of a printed stamp, and much more. This makes the evidence on personalization measures even more ambiguous, since studies apply personalization on various levels. With these highly heterogeneous results from previous trials in mind, we hence see a need for further research. Based on the existing evidence and methodological approaches, we expected positive effects of personalized study documents on participation. Yet, as previous research produced highly ambiguous results, we also expected rather incremental improvements that also depend on the context of a study.

## Research objectives

Our study aimed to determine the effects of a personalized address in an invitation letter on participation rate among GPs. This will add to the still ambiguous knowledge on personalization of study materials and in the long run might contribute to the development of methods aimed at increasing initial response and participation rates.

## Materials and methods

As part of the study “Cooperation networks of ambulatory health care providers: exploration of mechanisms that influence coordination and uptake of recommended cardiovascular care” (ExKoCare), we conducted an embedded randomized controlled trial on methods for recruiting general practitioners. ExKoCare is funded by Deutsche Forschungsgemeinschaft (DFG) and registered at the German Clinical Trials Register (DRKS, www.drks.de) under ID no. DRKS00019219. This study of 3 years aimed at recruiting a total sample of 40 general practitioner practices to explore coordination of cardiovascular care in the German states of Baden-Wuerttemberg, Rhineland-Palatinate and Saarland. In ExKoCare, practices and providers were asked to complete a questionnaire, a telephone interview and they were asked to support a patient study by handing out necessary study materials to patients that were randomly selected by them according to criteria defined by our research team. A detailed study protocol of the complete study has been published previously [17], hence the following description focuses on the recruitment trial. The conducted trial received approval from the Ethics Committee I of the Medical Faculty Heidelberg, Germany, under reference number S-726/2018. All methods were carried out in accordance with relevant guidelines and regulations and performed in accordance with the Declaration of Helsinki. All participants in the ExKoCare-study provided their informed consent. Separate informed consent for the trial reported in this manuscript was waived by the ethics committee.

### Study population and sampling

For the main study, we aimed at recruiting 40 GP-practices, and a priori we expected a low response rate of less than 10%. This estimate was based on the participation rate we experienced in previous, less complex studies requiring GPs to participate which varied between 6.0 and 18.0% [18]. For the trial reported in this paper, we did not plan a separate sample size, but calculated the statistical power post-hoc. We included a clustered, stratified sample of general practitioners from a total of 25 counties in the states of Baden-Wuerttemberg (roughly 11 million inhabitants, sampling in 10 out of 44 counties), Rhineland-Palatinate (roughly 4 million inhabitants, sampling in 13 out of 36 counties) and Saarland (roughly 1 million inhabitants, sampling in 2 out of 6 counties). The counties were chosen with regard to population density to ensure that rural and urban areas were represented likewise. All GPs needed to be based in an ambulatory practice, either as practice owner or employed doctors and needed to have a fax number, the latter still being the most widespread means of communication among physicians in Germany. Paediatricians were excluded because of the focus of the study. We aimed at contacting each general practice in the respective county. GPs were identified through the publicly available online physician’s databases in each of the three states [19–21].

As the study partly focused on collaboration within practices and hence required the practice to participate as a whole, we refrained from contacting multiple physicians in each practice when there were more than one. Instead, we aimed to identify the owner. Therefore, results were sorted out in our lists so that each practice was represented through one physician only. This led to a total sample of 1617 practices (Baden-Wuerttemberg: 912 practices, Rhineland-Palatinate: 596 practices, Saarland: 109 practices).

### Intervention

All practices were planned to receive an initial computer-generated fax letter with the request to indicate whether they were interested in the study or not and then send it back, also via fax. The letter was planned not to exceed one page, so that it could be read in a relatively short time and did not ask too much of the recipients. It contained a short introduction, a brief summary of the ExKoCare-project’s objectives and contents, workload to be expected as well as a mention of the reimbursement of 250 Euros participating practices received after completing the study. At the bottom of the page we included a separate paragraph with checkboxes to indicate one’s interest and our fax number with a request to send it back until a specified date (roughly 2 weeks away from the date of dispatch). The page furthermore included our institution’s letterhead, the names of the researchers involved and the logo of the funding organization DFG. It was signed in print by the project leader and a member of the research group who works as a GP. When a practice indicated interest in the study, they received the necessary documents by mail where they had the opportunity to either agree or decline to participate in the study.

Practices were randomized regarding the form of address: One version of the letter contained an individual, personal type of address (“Dear Dr. [NAME]”), the other version contained a generic type of address (“Dear Sir or Madam”). Recipients were blinded regarding group assignment, so they did not know whether they would receive a personalized letter or a generic one. If they sent the letter back and indicated interest in the study, further information and necessary documents for participation were sent out – these documents were identical for both groups. To participate, physicians had to mark another checkbox, sign a document indicating informed consent and mail the documents back to us.

During the randomization process, a total of 10 additional physicians in Baden-Wuerttemberg were removed from the list: 6 physicians belatedly proved to belong to practices already represented by other physicians, 3 physicians were accidentally removed because during the sorting process they appeared to belong to practices already present in the list, which later proved to be an error, and 1 practice was excluded because the practice owner was closely linked to our department and therefore had a high risk of bias. Randomization was carried out through sorting the practice list by random numbers generated via Microsoft Excel and assigning the practices to one type of letter accordingly. A total of 806 practices were selected as intervention group, receiving a personalized letter (455 in Baden-Wuerttemberg, 297 in Rhineland-Palatinate and 54 in Saarland) and a total of 801 practices were selected as control group, receiving a generic letter (447 in Baden-Wuerttemberg, 299 in Rhineland-Palatinate and 55 in Saarland) and contacted accordingly. Faxes were sent out online via an internal server which provided feedback on successful and unsuccessful transmissions. We sent out a total of 1607 letters, of which 1511 (94.0%) reached the respective practice. The complete procedure is also shown in a flow diagram derived from the CONSORT-template [22] which can be found as a supplement to this paper. A sensitivity analysis using the software G*Power showed that a sample size of 1511 allowed for effect sizes starting at w = 0.09 to be recognized, given one degree of freedom, an error probability of alpha = 0.05 and a power of 1-ß = 0.95.

### Outcomes and operationalization

We focused on three outcome variables: Response to our invitation, initial interest in the study, and indication of actual participation.

The dichotomous variable indicating response to our invitation was coded as “yes” whenever we received a reply to our letter, regardless whether the respondent showed interest in the study or not. Initial interest in the study then was indicated through the respective checkbox on the letter. In the analysis, interest was coded as “yes” when the checkbox indicating interest was marked and “no” when the one indicated no interest was marked. Responses without any marked checkboxes were counted as a missing value on the interest variable. Participation in the study was coded as “yes” when we received the necessary documents for participation filled-out accordingly and signed by the physician. The variable was coded “no” when the physicians declined participation on the documents or when the documents were not returned to us at all.

Independent variables we included were: Personalization of the letter (yes/no), sex (male/female), whether the recipient had a (doctor’s) degree (yes/no), the type of practice (group, single, ambulatory healthcare centre, other/unknown), the state the practice was based in (Baden-Wuerttemberg, Rhineland-Palatinate, Saarland) and the county the practice was based in (24 counties in total, 1 category per county).

### Statistical analyses

Statistical analyses were performed using IBM SPSS 24. Besides frequency analyses for the purpose of sample description, we focused on differences between intervention and control group regarding response rates, indication of interest in the study and eventual participation. Differences were analysed using odds ratio, relative risks (RR), Chi^2^-tests or, where necessary, non-parametric alternatives respectively. Alpha was set at 0.05. Additionally, we performed analyses to identify further, potential covariates of response and participation besides the type of address in the initial letter. Finally, binary logistic regression analyses (using binomial distribution and logit link function) were performed for all outcomes where the descriptive analyses showed significant effects.

## Results

Of the 806 personalized letters sent out in the intervention group, a total of 757 (93.9%) reached the respective practice. 49 (6.1%) dropped out because the letter could not be delivered by the system. Of the 801 generic letters sent out in the control group, a total of 754 (94.1%) reached the respective practice. 47 (5.9%) dropped out because the letter could not be delivered by the system. Table 1 shows a detailed breakdown by states.

**Table 1:** Numbers of fax letters sent out/delivered in the intervention and control group

Sample	State	Letters sent out	Letters delivered successfully
Intervention group	Baden-Wuerttemberg	455	426 (93.6%)
	Rhineland-Palatinate	297	283 (95.3%)
	Saarland	54	48 (88.9%)
	**Total**	**806**	**757 (93.9%)**
Control group	Baden-Wuerttemberg	447	425 (95.1%)
	Rhineland-Palatinate	299	278 (93.0%)
	Saarland	55	51 (92.7%)
	**Total**	**801**	**754 (94.1%)**

An overview over baseline characteristics of both samples in our study can be found in Table 2.

**Table 2:** Baseline characteristics of the intervention and control group

Characteristic	Intervention group (***n*** = 757) n (%)	Control group (***n*** = 754) n (%)
Sex		
*male*	489 (64.6)	468 (62.1)
*female*	268 (35.4)	286 (37.9)
(Doctor’s) degree	543 (71.7)	560 (74.3)
Type of practice		
*Group practice*	225 (29.7)	245 (32.5)
*Single practice*	399 (52.7)	373 (49.5)
*Ambulatory healthcare centre*	13 (1.7)	14 (1.9)
*Unknown/other*	120 (15.9)	122 (16.2)

Due to the high number of unknown cases, we decided not to take the type of practice into account for further analyses. Chi^2^-tests for independent samples showed that baseline characteristics did not differ significantly between intervention and control group.

Table 3 presents results on what percentage of the intervention and control group responded to our invitation at all, whether they indicated their interest in participation or not and what percentage eventually participated in the study.

**Table 3:** Response to the study invitation and actual participation numbers (intervention and control group)

Sample	State	Total respondents	Interested respondents (n (%))	Respondents participating (n (%))
Intervention group (*n* = 757)	Baden-Wuerttemberg	74	49 (66.2%)	15 (20.3%)
	Rhineland-Palatinate	33	22 (66.7%)	4 (12.1%)
	Saarland	5	3 (60.0%)	2 (66.7%)
	**Total**	**112**	**74 (66.1%)**	**21 (18.8%)**
Control group (*n* = 754)	Baden-Wuerttemberg	59	40 (67.8%)	11 (18.6%)
	Rhineland-Palatinate	25	18 (72.0%)	6 (24.0%)
	Saarland	6	6 (100.0%)	1 (16.7%)
	**Total**	**90**	**64 (71.1%)**	**18 (20.0%)**

The share of respondents per state varied between 10.4 and 17.4% in the intervention group and between 8.4 and 13.8% in the control group. Total response rate within intervention group was 14.8 and 11.9% within control group. Total participation rate in the study was 2.6%, with 2.8% in the intervention group and 2.4% in the control group, respectively.

Regarding our main outcome of interest, a possible effect of a personalized invitation letter on participation rate, both in total and by state we found a slight, but not significant relationship between the two variables with a relative risk to participate when receiving a personalized invitation (all states combined) of 1.17 [95%-CI: 0.62, 2.21]. There was also no significant association between personalized and generic invitations and interest in the study (RR = 1.17 [95%-CI: 0.82, 1.65]) and between personalization of the letter and whether physicians replied or not (RR = 1.28 [95%-CI: 0.95, 1.73]).

Taking into regard all respondents independent of the way they were addressed, there was a significant association between the state a practice was based in and whether we received a reply or not (two-tailed test, Chi^2^ (2) = 8.636, *p* < .05; Cramer-V = 0.076, *p* < .05; *n* = 1511). This also goes for an association between the respective county and whether we received a reply or not (two-tailed test, Chi^2^ (23)=43.051, *p* < .01; Cramer-V = 0.169, *p* < .01; *n* = 1511). The latter was also found separately for counties within the state of Rhineland-Palatinate alone (two-tailed test, Chi^2^ (11) = 22.121, *p* < .05; Cramer-V = 0.199, *p* < .05; *n* = 561).

Furthermore, the county a practice was based in was associated with whether physicians were interested in the study or not (two-tailed test, Chi^2^ (23)=36.690, *p* < .05; Cramer-V = 0.156, *p* < .05; *n* = 1509). Again, the same goes for counties within the state of Rhineland-Palatinate alone (two-tailed test, Chi^2^ (11)=24.718, *p* < .05; Cramer-V = 0.210, *p* < .05; *n* = 560).

Within the state of Saarland, we additionally found a significant association between sex and whether a physician participated in the study or not (two-tailed Fisher’s exact test, *p* < .05; *n* = 99).

Due to the effects of covariates on receiving a response at all in the descriptive analyses, we performed an additional, binary logistic regression analysis on influences on receiving a response. The results are shown in Table 4.

**Table 4:** Binary logistic regression analysis on factors influencing a response to the study invitation

Variable	Regression coefficient	Standard error	df	p	Exp(B)	95% confidence interval for Exp(B)	
						Lower threshold	Upper threshold
Personalized invitation	0.254	0.153	1	.095	1.290	0.956	1.739
State *(Ref.: Baden-Wuerttemberg)*			2	.015			
*Rhineland-Palatinate*	−0.475^**^	0.168	1	.005	0.622	0.447	0.865
*Saarland*	−0.372	0.335	1	.268	0.690	0.357	1.331
Sex: Female	0.076	0.157	1	.627	1.079	0.793	1.470
(Doctoral) degree	0.074	0.175	1	.673	1.077	0.764	1.518
Constant	−1.903^***^	0.198	1	.000	0.149		

^*^p < .05

^**^p < .01

^***^p < .001

The model itself was statistically significant (omnibus test of model coefficients: Chi^2^ (5)=11.918, *p* < .05; Hosmer-Lemeshow-test: Chi^2^ (8)=3.631, *p* > .05). However, the goodness-of-fit was low with Cox & Snell R^2^ = 0.008 and Nagelkerkes R^2^ = 0.014. The regression results supported our descriptive findings: Receiving a personalized letter slightly increased the risk of replying to our invitation compared to receiving a generic letter, but with *p* > .05 the effect could not be deemed statistically significant by our criteria, similar to the descriptive analysis. The effect of the state a physician was based in partly emerged in the regression analysis as well: There was a significantly lower chance of receiving a reply from physicians based in Rhineland-Palatinate compared to physicians based in Baden-Wuerttemberg (OR = 0.622, *p* < .01). For Saarland, no significant effects were found in comparison.

## Discussion

Recruitment of physicians, especially general practitioners, for studies is an important condition for health research. Low response and participation rates pose a major challenge to researchers and led to various attempts to conquer this. Our randomized trial examined the effects of personalized versus generic invitation letters on responses and participation among general practitioners in three German states.

Overall participation in the ExKoCare-study, in which this trial was embedded, was very low with a rate of 2.6%, but not lower than in other studies among ambulatory care physicians that imply more than the completion of a single questionnaire. This further supported the experiences from past studies on the challenge of recruiting sufficient GPs, as we mentioned in the introduction [1, 2, 4]. Over the past years, research showed that low or decreasing response and/or participation rates among physicians are not only found in Germany, but can be seen as an international phenomenon [23, 24]. In our case, an explanation for low participation might have been the study itself: The ExKoCare-study on coordination and uptake of recommended cardiovascular ambulatory care was purely observational with cross-sectional questionnaires and interviews, complemented by the task to hand out study materials to patients, but it still required a commitment of each practice over a time period of at least 1 to 2 years, as there were separate study phases. Selected practices were planned to participate over a total timespan of 3 years. Hence, practices may not have dealt any further with our invitation letter after they were informed about the workload to be expected and therefore did not react in any way or disproportionately disagreed to participate. This might have been fuelled further by the pandemic situation of 2020: At the time of the study and our trial, Covid-19 was highly prevalent in Germany with a substantial number of practices already facing an additional, unusually high workload in their day-to-day business and several of them functioning as specialized centres for (potential) Covid-19 patients.

We found only slight, statistically insignificant evidence for our hypothesis that personalization of invitation letters has a positive effect on participation in our trial. In this context, an important question is whether the observed difference can still be practically relevant. Relatively, the intervention led to a 2,8% participation rate in the intervention group vs. a 2,4% participation rate in the control group – a difference of 0,4 percentage points. In absolute numbers, these were 3 additional participants. Given our aim of recruiting 40 practices for the underlying ExKoCare-project, these 3 were indeed relevant for the project. However, in studies with larger populations and higher response rates, such a difference might be irrelevant.

Furthermore, our data showed slight, non-significant tendencies of personalization effects on physicians’ overall responsiveness. This is in line with the more recent studies presented in the introduction: As we mentioned there, personalization of invitation letters/e-mails showed either no effects at all or effects with a significance level of *p* > .05. However, effects still may have been present, but impeded by the complexity of the underlying study and the workload caused by the Covid-19 pandemic as discussed above. Especially the latter might have prevented practices from considering participation in any study at all already before our invitation arrived. As a result, total responses in both intervention and control groups might have been lowered to a number where statistical significance at a 5%-level does not occur anymore in our tests.

Even though we found no significant effects of personalized invitations, this does not necessarily imply that personalization as such is unable to increase participation, especially considering older research we presented which did show significant effects. A possible explanation for such mixed evidence are not only the numerous ways to personalize study documents of which we chose one single approach, but also the fact that personalization of any invitation or information documents in a study is but one approach to increase response rate and participation respectively. It has been argued that response is a result of several instruments being applied in a study, implying that underlying theories and approaches need to be implemented as a whole [25]. Hence, effectiveness of personalization might differ depending on the context.

The importance of context for response and participation of physicians was indeed suggested by the differences between states in the study. The odds to receive any response from physicians in Rhineland-Palatinate at all were significantly lower than within Baden-Wuerttemberg. Our institution is based in Baden-Wuerttemberg and physicians from within the state might have participated in studies conducted at our institution in the past or even have functioned as a research practice, hence for some a kind of trust might have been established. Trust is suspected to elevate respondents’ willingness to participate in studies [16]. Physicians from other states have probably heard about our institution but did not have any experiences participating in our studies, hence there was no established trust yet and their willingness to participate might have been lower than in Baden-Wuerttemberg. Furthermore, one could argue that Rhineland-Palatinate and Saarland both have universities with medical faculties active in research themselves and we do not know to what extent practices there were already occupied by studies performed by those institutions. However, this is not different from Baden-Wuerttemberg, since the state has a total of five medical faculties active in research, including our institution.

### Strengths & limitations

Our study bears several strengths & limitations. With 1511 physicians, we deemed the sample size sufficient – especially bearing in mind the results of the sensitivity analyses which showed that even smaller effects would have been found. However, as we already indicated in the discussion section, for the eventual response rates in both groups and the rather slight differences that occurred, our study might still have been underpowered and hence unable to show statistical significance. Including three states in the study allowed us to at least reduce possible effects emerging solely from state-related factors in analyses that focused on our total sample. However, the complexity of the underlying study and the then current pandemic in Germany may have led to biases as we elaborated on in the discussion section. Covid-19 posed a major challenge for health care providers, which probably has reduced the willingness to participate in scientific research. Furthermore, using only one approach of personalization of course only allows us to draw conclusions on that particular method and not on effects of personalized study documents on participation in general.

## Conclusions

Participation of general practitioners in studies is often low. Out of the manifold approaches to increase response to study invitations, personalizing invitations is a rather cost-efficient and fast method. Our study invitations with a personalized address proved to have a slightly positive, but not statistically significant effect on participation in our study compared to generic invitations. The results further showed the importance of the regional context on receiving a response at all. In conclusion, one personalization measure alone seems only slightly useful. However, this does not mean that personalization should not be applied: Existing measures are manifold, as the literature we presented showed. It seems useful to strive towards a common definition of what personalization encompasses and then complement personalization with other (successfully tested) interventions to enhance response rates. Each of these interventions may have little effects that add up to a total effect with a higher practical relevance. This demands further research on which combination of methods is feasible for studies including GPs and still manages to significantly increase study participation. On a larger scale, this should go beyond approaches for single RCTs, e.g. through an effort to create networks of GP-practices for research.

## Supplementary Information

**Additional file 1.**

## Data Availability

The datasets generated and/or analysed during the current study are not publicly available due to ongoing research but are available from the corresponding author on reasonable request.

## Abbreviations

DRKS: Deutsches Register Klinischer Studien [German Clinical Trials Register]
GP: General practitioner
