# Supplementary material for: Effects of personalized invitation letters on research participation among general practitioners: a randomized trial
Source: BMC Med Res Methodol. 2021 Nov 13;21:247. doi: 10.1186/s12874-021-01447-y (PMC8590365; doi:10.1186/s12874-021-01447-y)
Supplement: Supplementary file 1 — Additional file 1. [file 12874_2021_1447_MOESM1_ESM.docx]

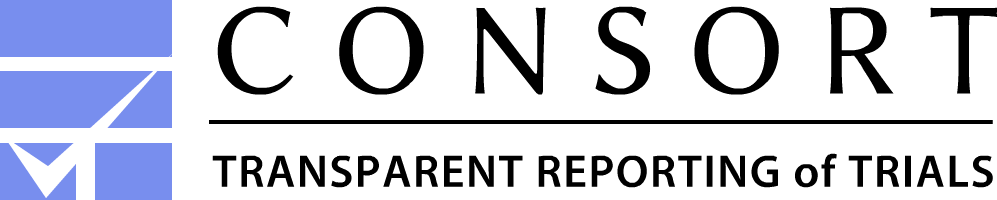


**CONSORT 2010 Flow Diagram for “Effects of personalized invitation letters on research participation among general practitioners: a randomized trial”**

## Follow-Up

## Enrollment

Allocated to intervention group (n=806)

♦ Received allocated intervention (n=757)

♦ Did not receive allocated intervention (fax unsuccessful) (n=49)

## Allocation

Allocated to control group (n=801)

♦ Received allocated intervention (n=754)

♦ Did not receive allocated intervention (fax unsuccessful) (n=47)

Randomized (n=1607)

Excluded (n=10)

♦  Not meeting inclusion criteria (n=6)

♦  Declined to participate (n=0)

♦  Other reasons (n=4)

Assessed for eligibility (n=1617)

[not applicable]

[not applicable]

## Analysis

Analysed (n=757)
♦ Excluded from analysis (n=0)

Analysed (n=754)
♦ Excluded from analysis (n=0)

This flow diagram is based on the CONSORT 2010 flow diagram under CC BY 2.0, available from:

<http://www.consort-statement.org/consort-statement/flow-diagram>
